# Target Speaker Detection with Concealed EEG Around the Ear

**DOI:** 10.3389/fnins.2016.00349

**Published:** 2016-07-27

**Authors:** Bojana Mirkovic, Martin G. Bleichner, Maarten De Vos, Stefan Debener

**Affiliations:** ^1^Neuropsychology Lab, Department of Psychology, University of OldenburgOldenburg, Germany; ^2^Cluster of Excellence “Hearing4all”Oldenburg, Germany; ^3^Department of Engineering, Institute of Biomedical Engineering, University of OxfordOxford, UK; ^4^Research Center Neurosensory Science, University of OldenburgOldenburg, Germany

**Keywords:** EEG, cEEGrid, around-the-ear EEG, mobile EEG, selective attention, speech decoding, cocktail party, attended speaker

## Abstract

Target speaker identification is essential for speech enhancement algorithms in assistive devices aimed toward helping the hearing impaired. Several recent studies have reported that target speaker identification is possible through electroencephalography (EEG) recordings. If the EEG system could be reduced to acceptable size while retaining the signal quality, hearing aids could benefit from the integration with concealed EEG. To compare the performance of a multichannel around-the-ear EEG system with high-density cap EEG recordings an envelope tracking algorithm was applied in a competitive speaker paradigm. The data from 20 normal hearing listeners were concurrently collected from the traditional state-of-the-art laboratory wired EEG system and a wireless mobile EEG system with two bilaterally-placed around-the-ear electrode arrays (cEEGrids). The results show that the cEEGrid ear-EEG technology captured neural signals that allowed the identification of the attended speaker above chance-level, with 69.3% accuracy, while cap-EEG signals resulted in the accuracy of 84.8%. Further analyses investigated the influence of ear-EEG signal quality and revealed that the envelope tracking procedure was unaffected by variability in channel impedances. We conclude that the quality of concealed ear-EEG recordings as acquired with the cEEGrid array has potential to be used in the brain-computer interface steering of hearing aids.

## Introduction

Complex every-day acoustic scenes such as the cocktail party (Cherry, 1953) are challenging listening situations, due to continuous masking of the target speech stream. In the presence of multiple sound streams, attending to the target speech stream is a demanding task, even for normal hearing individuals. Nevertheless, most individuals are capable of continuously perceiving auditory cues, such as pitch and timbre, from the target stream in the presence of masker streams. Consequently, more complex auditory objects are formed, and further utilized for selective attention to the target stream (Best et al., 2008; Moore and Gockel, 2012). On the other hand, it is known that hearing-impaired listeners have difficulties segregating acoustic streams in a complex auditory scene, which may be due to failure to receive the necessary acoustic cues or to deteriorated auditory object formation (Shinn-Cunningham and Best, 2008).

The preservation of acoustic cues is crucial for speech understanding in noise, with binaural cues being among the most imperative of these (Colburn et al., 2006). Nowadays, the preservation of binaural cues is implemented in hearing aids, providing an opportunity for directional hearing to the hearing impaired (Greenberg and Zurek, 1992; Marquardt et al., 2012; Thiemann et al., 2016). So far, however, these algorithms are beneficial only under the assumption that the desired speaker can be localized, which, in multi-speaker scenes, implies that the target speaker is either already known to the device or always found in a particular location.

It has been shown that the attended speaker can be identified from cortical recordings in complex auditory scenes. This has been shown with several recording technologies such as electrocorticography (Nourski et al., 2009; Zion Golumbic et al., 2013), magnetoencephalograpy (Ding and Simon, 2012; Akram et al., 2016), and EEG (O'Sullivan et al., 2014; Mirkovic et al., 2015). The procedure of choice for detecting the target speaker has been the envelope tracking algorithm (Pasley et al., 2012; O'Sullivan et al., 2014; Crosse et al., 2015; Mirkovic et al., 2015) which relates the envelope of speech to the neural signal. While the neural mechanisms underlying envelope tracking are a matter of debate, one hypothesis is that the activity of neuronal populations is synchronized to the acoustic features they are able to encode, resulting in temporal, as well as frequency coupling, of the neural networks with the temporal envelope of the acoustic stream (Shamma et al., 2011; Giraud and Poeppel, 2012; Ding and Simon, 2014; Vander Ghinst et al., 2016).

With this in mind, hearing aid users could benefit from a brain-computer interface (BCI)-guided selection of a to-be-attended speaker, which would in turn result in amplified processing of this stream, and hence facilitate listening. Next generation EEG technology, comprised of non-invasive surface sensors and miniature amplifier system, would be needed to acquire EEG signals in a concealed manner, with little hassle and high user comfort. At present, wireless mobile EEG systems are not small enough to fit behind the ear, but the quality of mobile EEG recordings acquired with head-mounted wireless EEG amplifiers can rival bulky, stationary lab systems (Debener et al., 2012; De Vos et al., 2014a,b; Zich et al., 2014), opening the possibility for combining BCIs and assistive technology. Recently, further progress has also been made with sensor technology. We developed flex-printed miniaturized EEG sensors, the cEEGrid sensor array, which positions ten electrodes in a C-shape around the ear and enables long-term EEG acquisition with little hassle or user discomfort (Debener et al., 2015). Currently, cEEGrids can be used in combination with a wireless, head-mounted mobile amplifier. Due to their size and position they can potentially be inconspicuously integrated with hearing aids, providing the potential for recording brain-states that could be used for natural, effortless steering of the hearing-aid. While we have previously shown that the acoustic envelope tracking method is robust even for the small number of optimally placed electrodes (Mirkovic et al., 2015), the question remains whether the cEEGrid locations around the ear allow for reliable data collection for this purpose.

In the present study, we asked whether concealed EEG acquisition with the cEEGrid sensor array enables identification of the attended speaker in a two-speaker scenario. To quantify performance of such a system, mobile wireless around-the-ear EEG (further referred to as ear-EEG) data were recorded concurrently with high-density state-of-the-art laboratory EEG (further referred to as cap-EEG) signals. This approach allowed us to replicate previous high-density cap-EEG envelope-tracking results, identify the possible loss in decoding accuracy that may be present in concealed ear-EEG recordings, and examine the impact of ear-EEG characteristics on target speaker identification.

## Materials and methods

### Participants

Twenty healthy, normal hearing, native German participants took part in this study (mean age 24.8 years, 8 male, 1 left-handed). Participants reported no past or present psychological conditions. One participant was excluded from the analysis due to technical problems and another was excluded due to poor performance in the attention task, as revealed by questionnaire results. All participants signed informed consent and were paid for their participation. The study was approved by the University of Oldenburg ethics committee.

### Paradigm

To investigate if the speech envelope tracking method, which is explained below, could be successfully applied to ear-EEG data we implemented a competitive speaker paradigm. This paradigm was the second of three tasks participants performed; results for the other two paradigms will be reported elsewhere.

The task was similar to the competitive speaker paradigm as used previously (O'Sullivan et al., 2014; Mirkovic et al., 2015). Stimulus presentation consisted of five blocks lasting 10 min each, with short breaks in between. Participants were instructed to attend to one of two simultaneously-presented speech streams, as indicated by the experimenter. Participants attended the same stream throughout the entire experiment, but were given a fresh instruction prior to each block. Participants were further required to keep their eyes open throughout the experiment. During the block, while the stimuli were being presented, a black fixation cross on light gray background was shown on the screen and participants were told to focus their gaze on it. During the breaks, participants were required to fill out a multiple choice questionnaire containing 10 questions pertaining to each speech stream. They were told to answer as many questions as they could but were further encouraged to continue attending only to one stream.

### Stimuli

Each of the two speech streams consisted of lesser-known fairy tales narrated in German language by one of two male professional speakers. Audio books featuring one of the speakers were retrieved from the German audio book website (Ohrka, 2012), while the material for the other speaker was obtained from the selection of audio books from Hering (2011). Prior to the experiment, the participants reported no, or very little, knowledge of these fairy tales.

For each audio track silent gaps longer than 500 ms were reduced to this length. Due to enunciation it occasionally happened that one speaker was perceived louder than the other, which led to differences in intelligibility between the two streams. To prevent the participants from involuntarily switching their attention during these periods, the stream with lower intensity was amplified using a weighting signal procedure. When the intensity difference between the two streams was high enough to cause a distraction, the amplitude of the weighting signal was calculated as the quotient of root mean square values for both streams within a 2 s long non-overlapping moving window. Otherwise, the amplitude of the weighting signal equaled 1. A first order low-pass Butterworth filter with 0.5 Hz cut-off frequency was applied to the weighting signal before it was used to modulate the audio stream, resulting in a similar intensity of both streams. As a result, both streams followed the rules of the natural conversation where one speaker would increase the intensity of their voice in the presence of other occluding sounds. A head-related transfer function (HRTF) from the corpus of Oldenburg University (Kayser et al., 2009) was applied to the streams to spatially distribute them. Impulse responses were recorded with the front hearing aid microphone in an anechoic environment at 0 degree elevation, −45 and 45 degree azimuth angle, and at a distance of 0.8 m from the speaker. Following our previous study (Mirkovic et al., 2015), the attended speaker location was kept constant within participants. Stimuli were processed with RME HDSP 9632 PCI Audio Interface and output presented to the participants using Tucker Davis Technologies programmable attenuators (PA5), via earphones (E-A-RTONE 3A) that participants were required to keep plugged in at all times during the entire experiment, except for the breaks. Visual instructions were presented on a screen located 1.3 m in front of the participant. Auditory stimuli and instructions were synchronized and presented to the participant using the Psychophysics toolbox (Brainard, 1997; Pelli, 1997; Kleiner et al., 2007) for Matlab, which also generated the marker stream with onset triggers.

### EEG recordings

**Figure 1:**
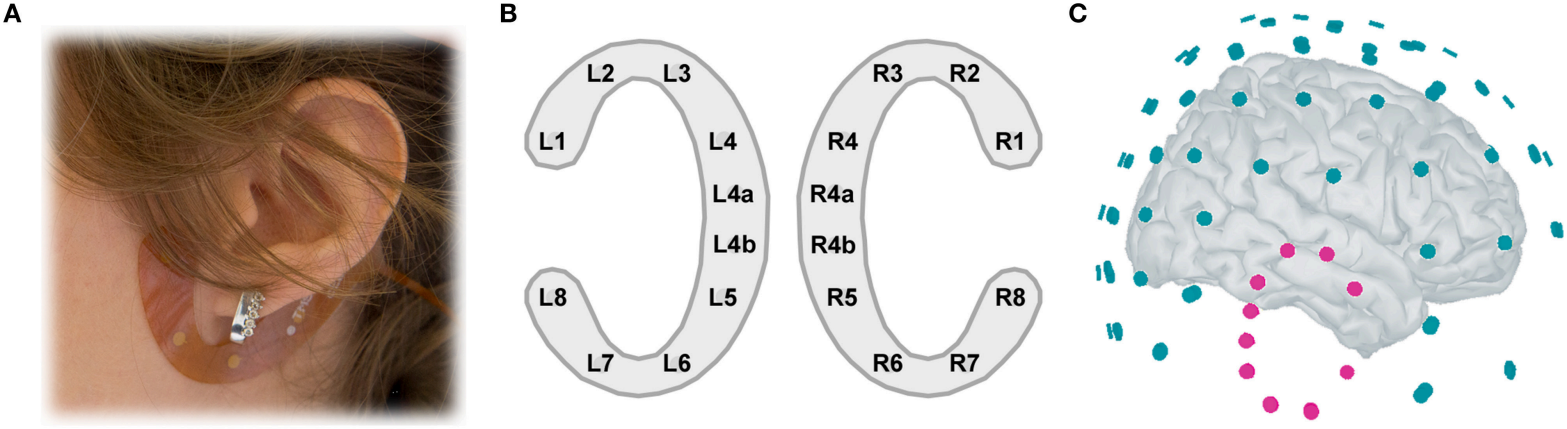
**(A)** cEEGrid placement. A C-shaped electrode array is fixated around the ear with a double-sided adhesive. **(B)** cEEGrid electrode layout. Each cEEGrid consists of 10 electrodes. Electrodes L1–L4 and L5–L8 around the left ear and R1–R4 and R5–R8 around the right ear measure the voltage between the respective electrode and the reference electrode R4b, while electrode R4a is the ground. L4b electrode was used in the offline analysis for rereferencing the data. To keep the layout symmetric, L4a electrode was excluded from analysis, leaving 16 channels in total, 8 around each ear. **(C)** Illustration of 84-channel cap-EEG and 16-channel ear-EEG 3D locations relative to each other, on a template brain. The left cEEGrid channels are hidden from view.

During the experiment, participants were seated in a dimly-lit sound-attenuated room in a comfortable chair. EEG was recorded with two electrode layouts simultaneously (Figure 1C). The ear-EEG consisted of two cEEGrids (Figures 1A,B) positioned around each ear of the participant. The cEEGrid is an array of ten flex-printed Ag/AgCl electrodes (cf. Debener et al., 2015). After skin preparation with an abrasive gel and alcohol a small amount of electrolyte gel (Abralyt HiCl, Easycap GmbH, Germany) was applied to the electrodes and the cEEGrids were placed with a double-sided adhesive around the ear. The two cEEGrids were connected to a wireless mobile 24-channel DC EEG amplifier (SMARTING, mBrainTrain, Belgrade, Serbia) positioned at the back of the head. Ear-EEG data were recorded with 24 bit resolution and 500 Hz sampling rate; positions R4a and R4b served as ground and reference, respectively. Signals were wirelessly transmitted to a recording computer through Bluetooth connection.

The second electrode setup consisted of 84 channels from an equidistant 96-channel EEG cap, provided from Easycap GmbH, Germany. Twelve electrodes around the ears could not be used due to the positioning of the cEEGrids. The high-density EEG cap was connected to 16-bit BrainAmp amplifiers (Brain Products, Gilching, Germany). The data were referenced to the nose tip and collected with a sampling rate of 5000 Hz, with an analog filter setting of 0.0153–250 Hz. After placing the high-density EEG cap the SMARTING mobile EEG amplifier was attached to the back of the participant's head with a rubber band over the cap, keeping participants' comfort in mind. Additionally, before and after the experiment the impedance on all cap-EEG and ear-EEG electrodes was recorded.

**Figure 2:**
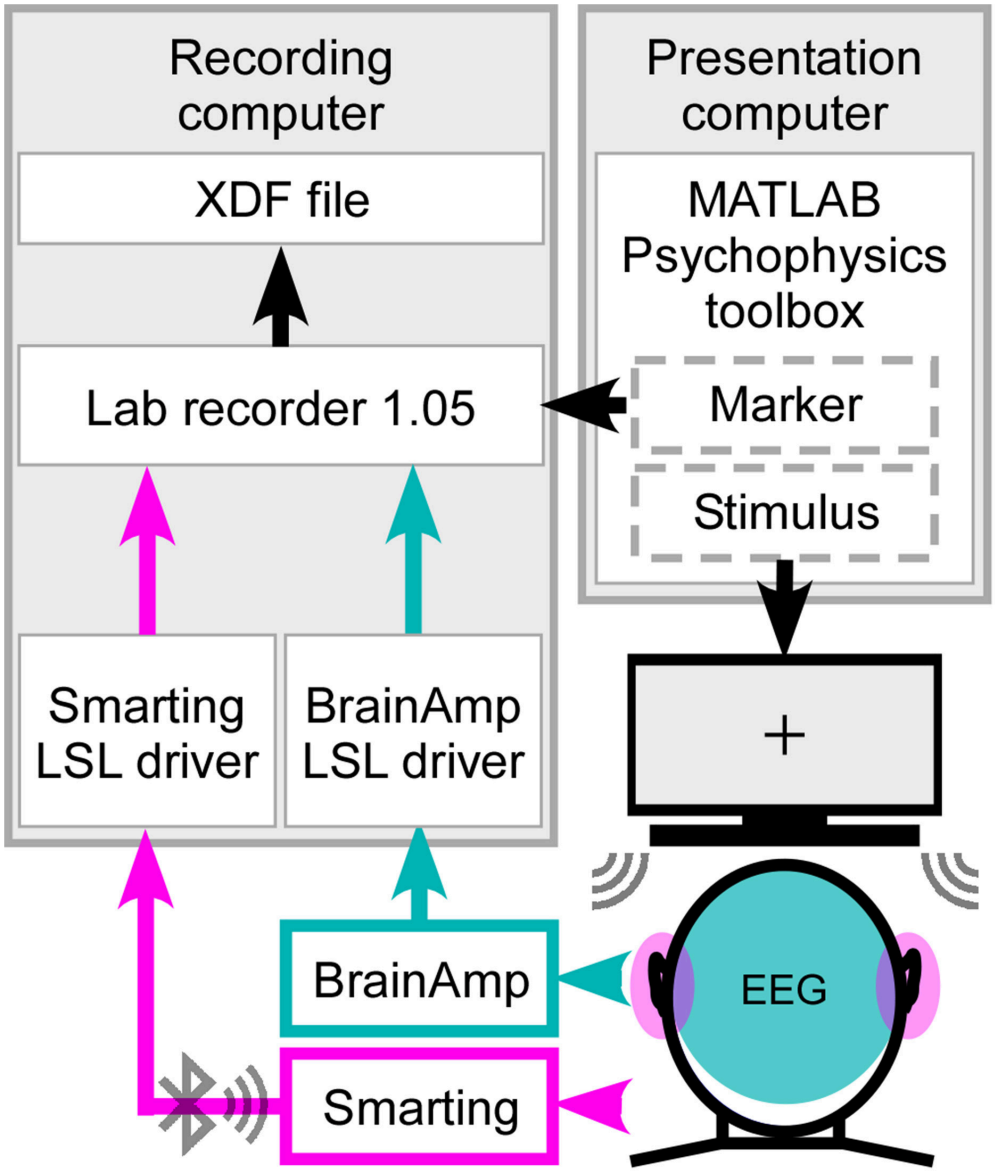
**Synchronized streaming of two separate EEG systems**. For stimulus presentation the Psychophysics toolbox was used to present the sound to the participant and send the event markers to Lab recorder. EEG was recorded with one cEEGrid located around each ear (head, pink) and high-density cap with 84 effective channels (head, cyan). Using the SMARTING mobile amplifier the ear-EEG signal was wirelessly sent to the recording computer, while the cap-EEG signals were recorded using the Brain Amp amplifiers with a physical connection to the computer. Through Lab recorder, a marker stream and both EEG streams were joined into one output file in extensible data format (.xdf).

The ear-EEG and cap-EEG signals as well as the marker stream sent from the Psychophysics toolbox were integrated using the Lab Recorder software (Figure 2) from the Lab Streaming Layer (LSL) package, a system for unified, time synchronized measuring. For this purpose a BrainAmp LSL driver, with a redesigned resampling filter performing the downsampling from 5000 to 500 Hz, was used for recording, as well as the Smarting Streamer software, which features a built-in LSL driver.

### Latency shift

Due to the hardware characteristics of both amplifiers and software filtering within the Brain Amp LSL driver, a latency shift between the two EEG systems could be expected. In order to verify good temporal precision of the set-up, sinusoidal signals were added to the beginning and end of 10-min long audio signals. The stimuli were presented via Psychophysics toolbox and the output of one eartone transducer was acquired with SMARTING and BrainAmp amplifiers and the signal from both combined into one output file by Lab recorder. This procedure revealed a constant latency shift of 50 ms between the two EEG streams, which was compensated for off-line.

### EEG and speech envelope preprocessing

EEG data were analyzed off-line using EEGLAB version 13.4.4b (Delorme and Makeig, 2004). Following the base-line correction, the cap-EEG data were re-referenced to the common average and filtered between 2 and 8 Hz using the Hann windowed FIR filter (low-pass filter of order 100, high-pass filter of order 500). Afterwards the continuous data were downsampled to 64 Hz and epoched in consecutive 60 s intervals, resulting in *N*_*t*_ = 50 trials per subject. Likewise, ear-EEG data were baseline corrected and re-referenced to algebraically linked mastoids (i.e., L4b and R4b). In order to keep the cEEGrid layout symmetrical, L4a electrode was removed from further analysis. As with the cap-EEG recordings ear-EEG data were then filtered from 2 to 8 Hz, downsampled to 64 Hz and divided into fifty 60 s non-overlapping trials. The envelopes of both audio streams were computed as the absolute value of the Hilbert transform, low-pass filtered below 8 Hz and downsampled to 64 Hz (cf. Mirkovic et al., 2015).

### Envelope tracking

Attended speaker in the competitive speaker paradigm can be identified using the envelope tracking method, a speech decoding algorithm (O'Sullivan et al., 2014). Provided that both envelopes of the presented speech streams are available, a multivariate linear regression model can be trained to associate the attended stream with corresponding EEG recordings. Once trained, the model is used to estimate the envelope of the attended speech stream from the EEG recordings without the prior knowledge on the stream itself.

As previously mentioned, the model is trained on EEG recordings and the envelope of attended speech using a multivariate linear regression. The lag between the EEG signal and the auditory stream has to be accounted for in the model in order to temporally align them. A time lag between the stimulus presentation and the corresponding neural processing is taken into account by time shifting EEG recordings relative to the stimulus **S***(*1x*t)*. Time shifts of different durations were simultaneously introduced through zero-padding of the EEG data matrix, resulting in several time-shifted EEG matrices which were subsequently concatenated into a data matrix **R**. The model, represented by the decoder **g**, which would associate matrix **R** to stimulus **S**, was trained for each 60 s trial using multivariate linear regression. Furthermore, the decoder was trained separately for cap-EEG and ear-EEG data.

$${\text{g}}_{l}={\left({\text{R}}_{l}{}^{T}{\text{R}}_{l}+\lambda_{l}\text{M}\right)}^{-1}{\text{R}}_{l}{}^{T}{\text{S}}_{l},l\in \left\{\text{cap},\text{ear}\right\}$$

The regularization matrix **M** used here is similar to that used in Mirkovic et al. (2015). Due to the differences in channel number and spatial distribution between cap- and ear-EEG, the regularization parameter λ_*l*_ differed for the two systems (λ_cap_ = 1000, λ_ear_ = 1). For the ear-EEG data, where the number of channels is lower, the risk of overfitting is also low, resulting in a smaller value for the regularization parameter. λ_cap_ and λ_ear_ were estimated, separately, by grid search, resulting in maximal decoding accuracy for cap-EEG and ear-EEG, respectively. This resulted in a total of N_*t*_ = 50 decoders for each EEG setup which could be assessed using the leave-one-out validation procedure. For this purpose we averaged N_*t*_-1 decoders and estimated the performance of the averaged decoder by applying it to the time-shifted EEG recordings (**R**) of the remaining trial, separately for both systems. In this way, for each trial *k* we obtained two estimated attended speech envelopes (further referred to as EASE), one resulting from cap-EEG and the other from ear-EEG.

$$\begin{array}{l} {\text{EASE}}_{l}^{\left(k\right)}={\left(\frac{1}{N_{t-1}}\sum_{i=1,N_{t};i\neq k}g_{l}^{\left(i\right)}\right)}^{T}R_{l}^{\left(k\right)},\\ \;k=1,N_{t},l\in \left\{\text{cap},\text{ear}\right\} \end{array}$$

Note that the EASE is not a close replica of the envelope of the original audio stream. Nonetheless, it is expected to correlate more closely with the envelope of the supposed to be attended stream (producing N_*t*_ correlation coefficients **r**_**a**_) than with the envelope of the supposed to be unattended stream (**r**_**u**_). By comparing the coefficients **r**_**a**_ and **r**_**u**_ an informed decision can be made on which stream the participant was attending. Hence, we classified EASE for each of the N_*t*_ trials as attended or unattended, based on the higher of the two correlation values for corresponding trial. The decoding accuracy was calculated as the percentage of accurately classified trials, resulting in two values for each participant, DA_cap_ and DA_ear_.

### Optimal time lag

To find the time-lag interval resulting in optimal decoding accuracy we analyzed the pattern of correlation values for a variety of time-lag intervals. To this end we used a 45 ms moving time lag window with 30 ms overlap in a −115 to 620 ms time-lag range, and calculated EASE for each window. We determined the correlation of EASE with original envelopes and averaged the Fisher's z-scores of resulting *r*-values over trials and participants. The average z-scores were then back-transformed into *r*-values. This analysis was performed for both cap-EEG and ear-EEG data separately. A common optimal time-lag interval for both layouts was determined as the interval in which the difference between *r*-values for the attended and unattended streams was greatest.

### Scalp data verification

Prior to proceeding to the analysis of ear-EEG data we verified the measurement and analysis procedure by comparing the decoding accuracy achieved with cap-EEG (DA_cap_) with the accuracy achieved by reanalyzing our already published data (Mirkovic et al., 2015). To this end, the regularization parameter for both datasets was kept constant at λ_cap_ and the time-lag interval was determined by the optimal time lags for the current data. The number of channels in the data from the previous study was reduced to 84 by selecting the corresponding channels. To analyze the influence of channel reduction, decoding accuracy was recalculated on the data from the previous study using the same parameters and the full 96-channel layout.

### Overall performance

Decoding accuracy is an informative but sparse measure of performance. Keeping in mind that it is a classification based on the correlation of EASE and original sound envelopes, a complementary measure of participants' performance may be related to the *r*-values themselves. For each 60 s trial we therefore calculated the difference between z-transformed *r*-values for attended and unattended streams and averaged them for each participant. Therefore, the performance score P was given as:

$$P=Z^{-1}\left\{\frac{1}{N_{t}}\sum_{i=1}^{N_{t}}\left(Z\left\{r_{a}(i)\right\}-Z\left\{r_{u}(i)\right\}\right)\right\}$$

where *Z* stands for the z-transform operator, *N*_*t*_ the number of trials and **r**_**a**_/**r**_**u**_ are vectors comprised of *N*_*t*_ = 50 correlation coefficients between the EASE and attended (a)/unattended (u) speech envelope.

The differences in *r*-values reflect both the enhancement of the attended stream and/or the suppression of the unattended stream and are consequently convenient estimates of participants' performance. This procedure provided two performance scores for each participant, one for the ear-EEG data (P_ear_) and one for the cap-EEG data (P_cap_). Providing that the information necessary for speech decoding is indeed present in ear-EEG data, the ear-EEG performance scores should correlate with cap-EEG derived performance scores. To investigate this relationship, we calculated the corresponding Spearman rank correlation.

### Influence of electrode position on speech decoding

To investigate the influence of electrode placement we defined six electrode clusters from cap-EEG data keeping the cluster size identical to the number of ear-EEG channels (16). The electrode clusters were defined as: (1) a frontal scalp area symmetric about the sagittal plane, (2) a posterior scalp area symmetric about the sagittal plane, (3) a central clustering around the Cz electrode, (4) left and right temporal areas in close proximity to cEEGrids, (5) a frontal and posterior area symmetric about the sagittal plane, and (6) wide scalp coverage. Furthermore, we looked into the possibility of speech decoding with the right cEEGrid only, in which case the data were not re-referenced.

### Influence of ear-EEG properties on speech decoding

To investigate the significance of electrode impedance on the ear-EEG recordings and correspondingly on overall performance (P_ear_), we made a performance-based median split of participants and performed an independent samples *t*-test for each cEEGrid electrode impedance between the bad and good performers. Hereby, the impedance measurements were performed for 17 out of 18 previously analyzed participants. Impedance values for the remaining participant were absent due to technical reasons. The impedance values were calculated for each electrode as the mean of the impedances measured before and after the experiment. Furthermore, in order to investigate the difference in adherence of upper cEEGrid electrodes, which may be reflected in impedance values on cEEGrid electrodes located above the ears (R2–R3, L2–L3), we performed a paired sample *t*-test between these and the bottom cEEGrid electrodes (R6–R7, L6–L7), where the cEEGrid could be more firmly attached. Keeping the median split we also analyzed EEG frequency band activity for each ear-EEG channel. For this purpose we defined the following frequency bands: 1–3, 4–7, 8–12, 13–30, 31–80, 81–150, 151–200, and 201–250 Hz. The corresponding band power values were submitted to independent samples *t*-tests.

We further analyzed the influence of the angle at which the cEEGrid was placed on a participant's head. After EEG acquisition an infrared 3D digitizer was used to record the position of all cap and cEEGrid electrodes (Xensor electrode digitizer, version 5.0.0, ANT Neuro, The Netherlands) as well as individual head shape coordinates. We used the digitized electrode positions to project the four cap electrodes positioned in line with Cz (following the frontal plane) onto the sagittal plane. The least square regression line of these projections was defined as the “head axis.” Next, the upper two electrodes of the left and right cEEGrids (L2–L3 and R2–R3) were projected onto the same plane. The mean positions between the projections of upper left/upper right cEEGrid electrodes were calculated (resulting in positions L_23_ and R_23_). The same was done for the bottom electrodes, resulting in two more positions on the sagittal plane (L_67_ and R_67_). The axes of cEEGrids were defined by positions L_23_ and L_67_ for the left cEEGrid and R_23_ and R_67_ for the right cEEGrid. Statistical analysis was performed on cEEGrid axis deviation from head axis using the independent samples *t*-test between good and bad performers.

## Results

### Questionnaire analysis

One participant with a questionnaire score lower than the designated threshold of 50% of correctly answered questions pertaining to the attended story was excluded from further analysis. The remaining participants answered on average 86.1% of the questions referring to the attended story correctly (ranging from 68 to 100%), while they could not answer questions to the unattended story (< 0.1% correct answers on average).

### Optimal time lag

**Figure 3:**
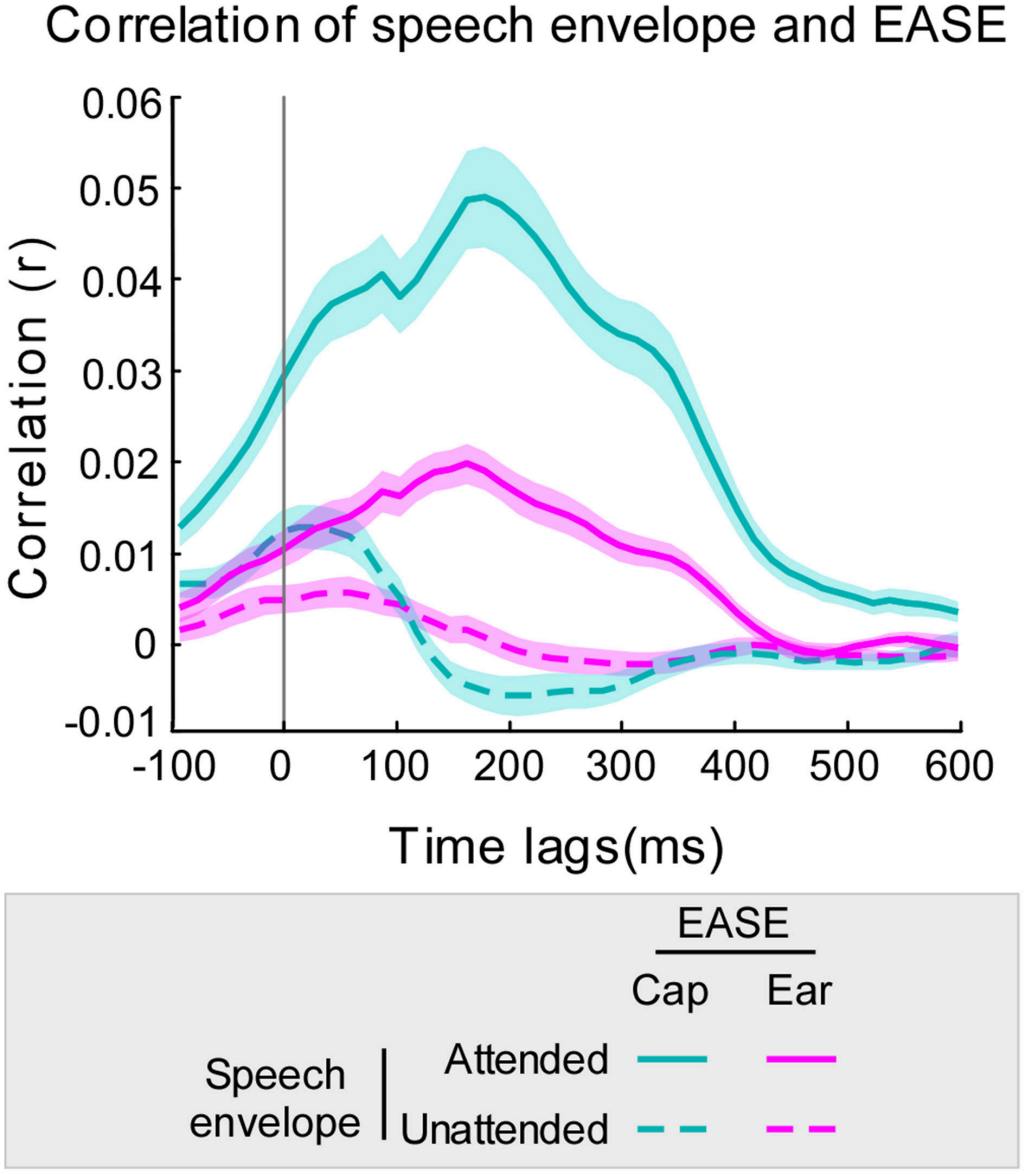
**Correlation of EASE and envelopes of audio streams using 45 ms overlapping time lag windows**. Correlation of the audio stream envelopes with EASE_cap_ is illustrated in cyan and with EASE_ear_ in pink (attended stream in a solid line, and unattended stream in a dotted line). The cyan/pink shaded areas represent the standard error of the mean for cap-EEG and ear-EEG recordings respectively.

The time-lag analysis of *r*-values revealed the optimal lag intervals for speech decoding to be between approximately 140 and 200 ms (Figure 3). Furthermore, at all time lags the mean correlation of EASE with the attended stream was higher than the mean correlation of EASE with the unattended stream. This was the case for both cap-EEG and ear-EEG recordings. As hypothesized, a similar lag pattern was noticeable for the correlations of speech envelopes with EASE_cap_ and EASE_ear_. The Pearson's correlation of lag patterns resulting from the comparison of attended speech envelope with EASE_cap_/EASE_ear_ was strong (*r* = 0.98, *p* < 0.001), and the same applied for lag patterns resulting from comparison of unattended speech envelope with EASE_cap_/EASE_*ear*_ (*r* = 0.88, *p* < 0.001). However, EASE_ear_ was not as strongly correlated with the envelopes of audio streams as EASE_cap_, reflected in noticeably lower correlation values as confirmed by paired-samples *t*-test [*t*_(46)_ = 13.32, *p* < 0.001, *d* = 1.427].

### Scalp data verification

**Figure 4:**
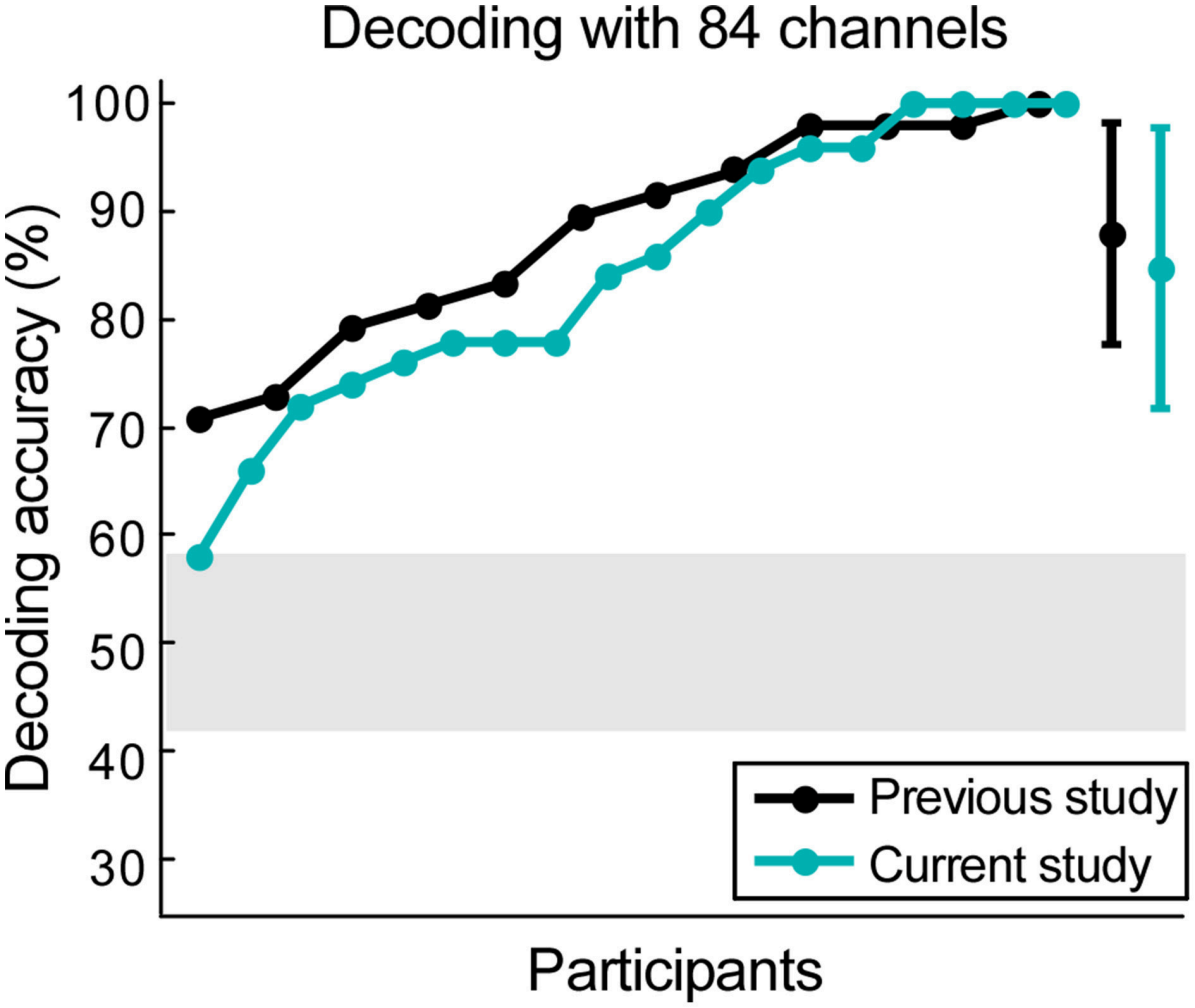
**Scalp data verification**. The data from our previous study was reanalyzed, keeping only 84 channels that were currently available. Results from data collected from 12 participants in the previous study are illustrated in black, while the results from 18 participants in the current study are illustrated in cyan. The gray shaded area represents chance level and the error bars signify the standard deviation for both datasets.

The 84 available scalp channels produced a decoding accuracy of 84.78% when the optimal time lags were used. By reanalyzing the data from our previous study (Mirkovic et al., 2015) with the same 84 channels an average decoding accuracy of 88.02% was found. An independent-samples two-tailed *t*-test comparing decoding accuracy resulting from both datasets did not reveal a significant difference [*t*_(28)_ = 0.73, *p* = 0.473, Figure 4).

In both cases all participants performed above chance level. Maximum decoding accuracy was reached by one participant in the previous study (Mirkovic et al., 2015) and four participants in the current study. Using the same decoding parameters and all 96 channels from the previous study revealed a mean decoding accuracy of 89.23%. A paired-samples *t*-test revealed no significant difference between this result and the decoding accuracy achieved with the same participants and the 84 channel layout [*t*_(11)_ = −1.13, *p* = 0.281].

### Overall performance

**Figure 5:**
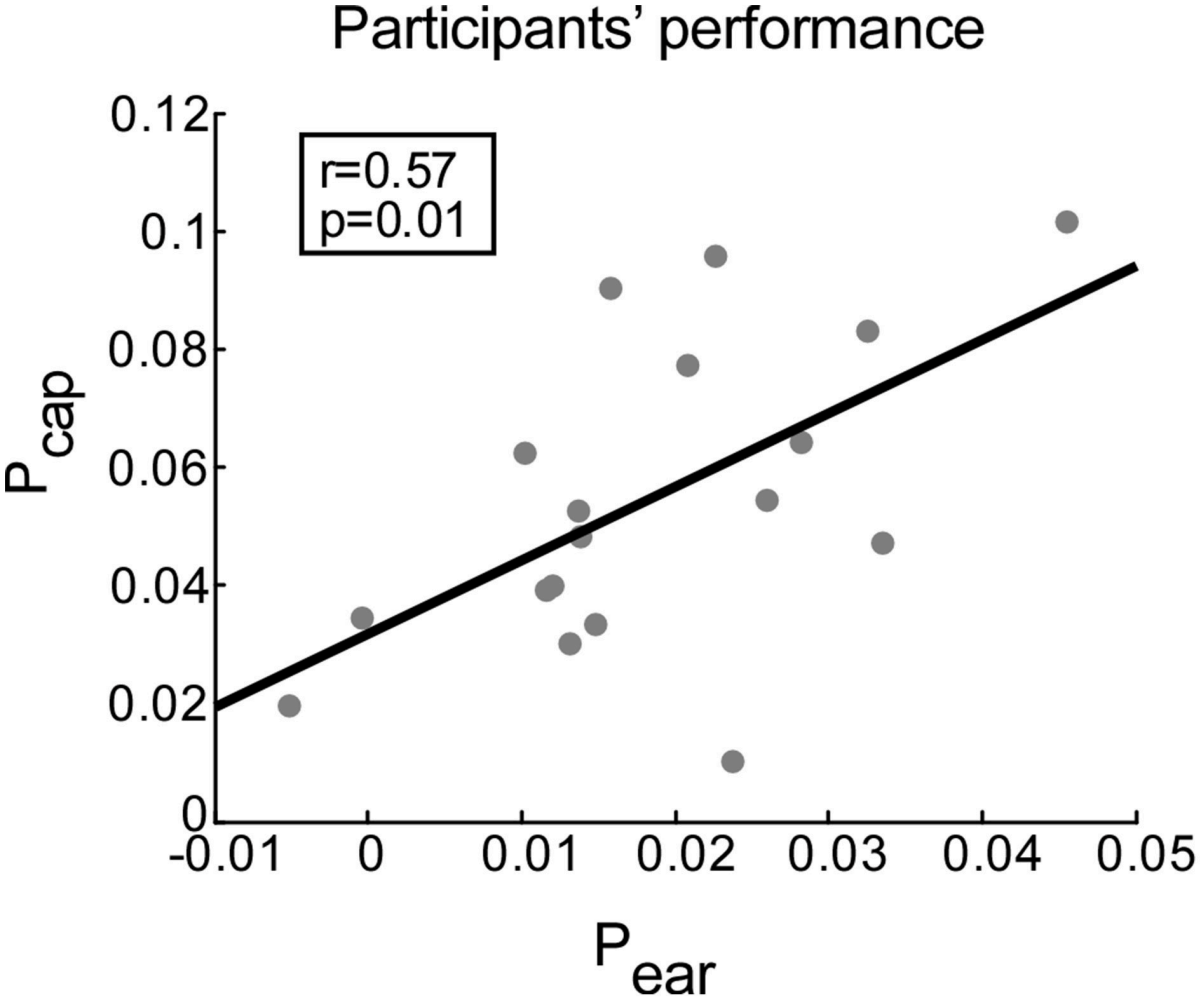
**Correlation between performance scores achieved with cap-EEG and ear-EEG recordings**. Performance is measured for each participant as the average difference between the correlation of EASE with the attended (r_a_) and unattended stream (r_u_) envelopes. Gray dots represent participants. The least squares regression line is shown in black.

The performance scores for ear- and cap-EEG data can be seen in Figure 5. A positive correlation between the performance scores obtained by ear-EEG and cap-EEG data was evident (*r* = 0.57, *p* = 0.012). Subsequent paired-samples *t*-test showed that the performance score was significantly lower for ear-EEG as compared to cap-EEG data [*t*_(17)_ = 7.07, *p* < 0.001, *d* = 1.77, Figure 5].

### Influence of electrode position on speech decoding

**Figure 6:**
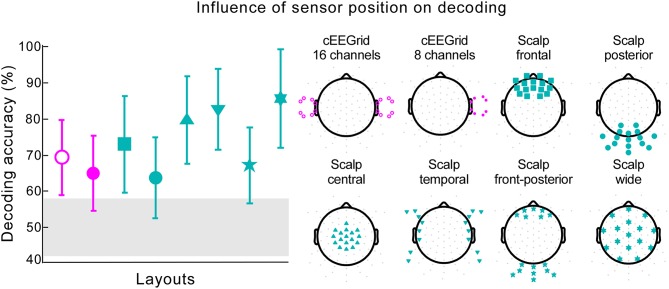
**Influence of sensor position on decoding**. The decoding accuracy achieved with various electrode layouts (illustrated on the right) is shown on the left. Markers on each layout on the right indicate the channels used in the decoding algorithm. The marker shape on each layout on the right corresponds to the respective decoding accuracy result shown on the left. Pink markers signify cEEGrid channels and cyan markers cap channels. The error bars represent the standard deviation across participants. The gray shaded area represents chance level.

We explored the role of different electrode positions and spatial distributions for decoding accuracy. When information from only one cEEGrid was used, decoding performance reached 64.88% with five participants performing at or below chance level (Figure 6, cEEGrid 8 channels). Data collected from two cEEGrids, one placed around each ear, resulted in decoding accuracy of 69.33% (Figure 6, cEEGrid 16 channels). Two of the 18 participants performed below chance level. By reanalyzing our previously published cap-EEG data using around-the-ear channels only, with reference at the nose tip, we achieved a decoding accuracy of 76.22%. This was not significantly different than decoding with two cEEGrids [independent samples *t*-test, *t*_(28)_ = −1.564, *p* = 0.129]. Further, six different clusters of 16 cap channels were considered. A cluster with 16 channels covering only posterior scalp sites resulted in lowest performance, which was 63.78% (Figure 6, Scalp posterior). Compared to layouts covering frontal and/or posterior sites, clusters that included central coverage, where auditory brain-electrical activity is usually most pronounced, resulted in higher decoding accuracies. Here, the information from the widespread electrode distribution (Figure 6, Scalp wide) resulted in the best decoding accuracy of 85.67%, which was not significantly worse than decoding for all 84 channels, as reported by a paired-samples *t*-test [*t*_(17)_ = 0.22, *p* = 0.825]. A paired-samples *t*-test showed that performance was significantly better for the wide coverage than for a focal central coverage [*t*_(17)_ = 4.85, *p* < 0.001, *d* = 0.457], which resulted in the mean of 79.78%. In both cases only one participant performed below chance and for the wide central coverage, maximum decoding accuracy was achieved for two participants. Data collected from the 16 cap channels closest to the cEEGrids (Figure 6, Scalp temporal) resulted in decoding accuracy of 82.78%.

### Influence of cEEGrid properties on speech decoding

**Figure 7:**
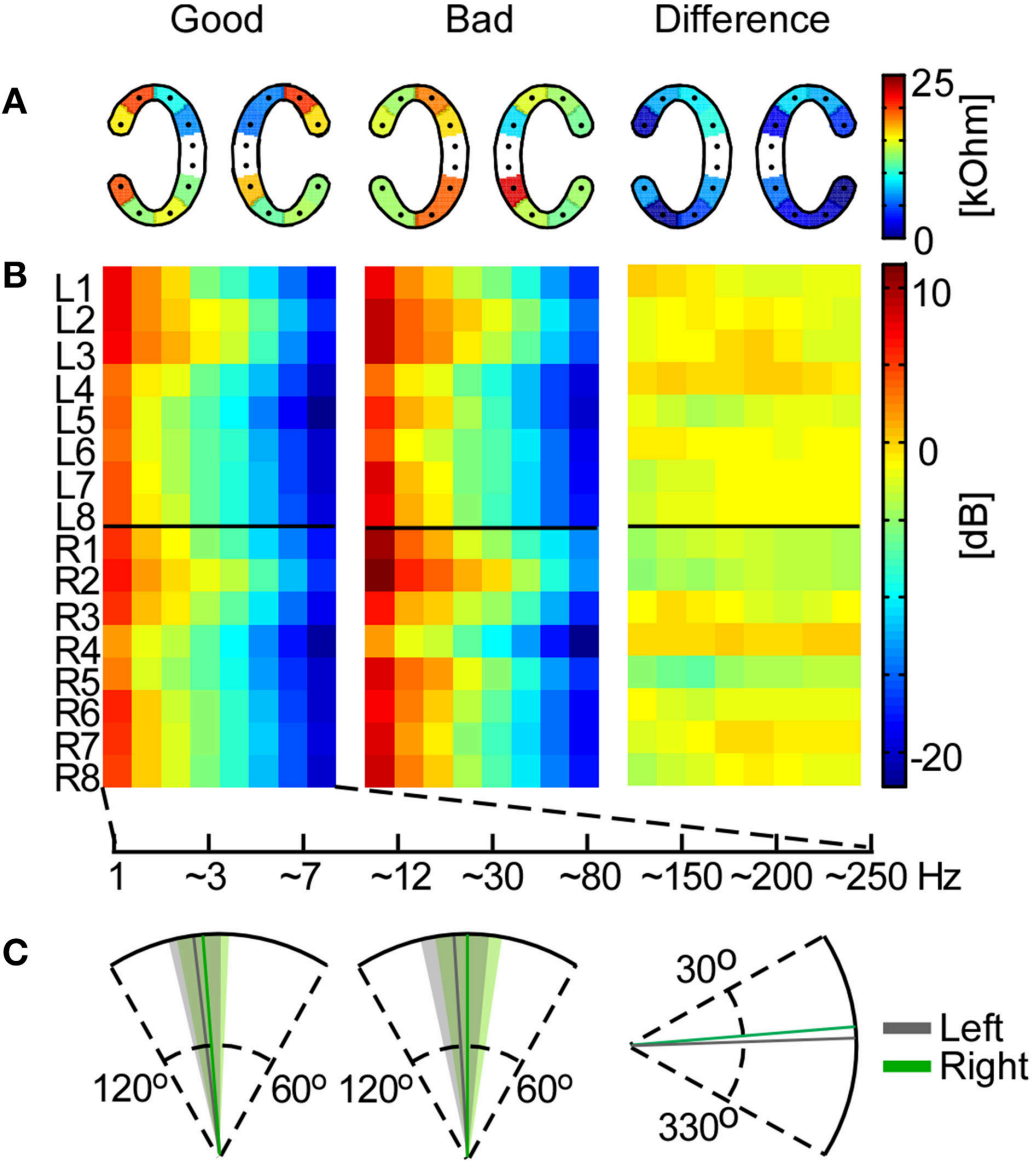
**Influence of cEEGrid properties on participants' performance**. In the left and middle plots the mean across good and bad performers, respectively, is shown for each analysis. **(A)** Mean cEEGrid electrode impedances across participants. The right plot shows the absolute difference between the two group means. **(B)** Frequency band analysis for all cEEGrid electrodes. On the right is the difference between the group means. **(C)** The cEEGrid placement angle with standard deviation (left cEEGrid is shown in gray, right cEEGrid in green). On the first two plots the head axis is considered to be at 90°. On the third plot, the difference between the group means is measured from 0°.

The measured impedances on cEEGrid electrodes ranged from 0.6 to 83.6 kOhm with a median value of 13.5 kOhm. For each cEEGrid electrode we calculated the median impedance over participants. Based on the median ear-EEG performance (P_ear_) we split the participants into good and bad and compared the impedance on each electrode for these two groups. According to independent samples *t*-tests there was no significant difference (*p* > 0.1) between good and bad performers (Figure 7A). Furthermore, paired sample *t*-tests between the upper and lower channels (R2–R3 vs. R6–R7 on the right cEEGrid; L2–L3 vs. L6–L7 on the left cEEGrid) did not reveal any significant difference (*p* > 0.05), even though the electrode-to-skin adherence conditions on upper and lower sites may have differed.

Furthermore, the frequency band analysis showed that in both good and bad performers the spectrum resembled typical EEG characteristics with low energy in high-frequency bands (Figure 7B). Again, systematic differences between good and bad performer groups were not detected (independent samples *t*-tests, *p* > 0.05), either from a particular channel or for a particular frequency band, which suggests that ear-EEG signal quality differences between individuals did not contribute significantly to decoding performance.

Finally, we analyzed the angle of the cEEGrid placement, which was individually adjusted to fit each participant's ear anatomy. The mean angle for both groups, and the mean angle difference between groups, is illustrated in Figure 7C. The cEEGrids were placed parallel to each other (*SD* = 6.6°) and parallel to the head axes (right cEEGrid *SD* = 7.8° and left cEEGrid *SD* = 7.2°) in all participants.

## Discussion

Aiming toward our ultimate goal of inconspicuous and hassle-free long-term EEG acquisition, we investigated the potential of ear-EEG for decoding the attended speaker in a competitive speaker paradigm. While participants were presented with two concurrent speech streams, two separate EEG recording systems were used for signal acquisition, a high-density cap-EEG system and a wireless head-mounted EEG system, the latter of which was combined with the cEEGrid sensor technology. Based on previous ear-EEG work in our lab (Bleichner et al., 2015; Debener et al., 2015) we hypothesized that the cEEGrid ear-EEG system would capture brain activity relevant for attended speaker decoding.

### Cap-EEG performance

We first validated the cap-EEG signal quality by comparing the decoding accuracy of the present study with our previous work (Mirkovic et al., 2015). Unlike in our previous experiment, which used one male and one female speaker, in the current experiment the audiobook stories were narrated by two male speakers, which resulted in a more demanding task for the participant. Another difference was the use of a HRTF to implement spatial cues, whereas only interaural level differences were used before. HRTF supports a spatial release from masking, which is an essential part of real-life multi-speaker scenarios and is beneficial for separating the target speaker from other speech streams (Arbogast et al., 2005; Colburn et al., 2006). A more subtle difference between studies was the use of the cEEGrids, which limited cap-EEG recordings to 84 channels. In order to address this latter discrepancy we removed the around-the-ear cap channels from our previous dataset and performed the same speech decoding analysis for both studies. The resulting decoding accuracy was very similar and significant differences could not be observed between studies. Accordingly, we conclude that identification of the attended speaker in a competitive speaker scenario from multi-channel EEG is a robust observation, even if the audio streams used and the spatial features implemented differ. The speech decoding method applied appears to be very robust and largely unaffected by characteristics of individual speakers. The comparison between the previous and the current cap-EEG results also suggests that the presence of a head-mounted wireless EEG system, and use of two cEEGrids, did not interfere with the signal quality obtained from cap-EEG recordings.

### Ear-EEG performance

While the first cEEGrid validation study (Debener et al., 2015) demonstrated that this type of ear-EEG setup captures various brain signals, such as auditory evoked potentials, P300 event-related potentials to target events, and resting EEG alpha activity, we show here that more complex processes, such as connected speech perception in a challenging listening situation, leave their traces in cEEGrid EEG recordings. Across subjects, ear-EEG and cap-EEG decoding performance was correlated, which favors the claim that cEEGrids capture at least a reasonable part of the cortical activity that enables attended speaker decoding. The same conclusion can be drawn from the time-lag analysis. A very similar time-lag pattern for the EASE correlation with both streams was present for cap-EEG and ear-EEG, which supports the claim that both results are driven by the same process. Evidence in favor of an attention-related cortical signature is further provided by the fact that the maximum performance occurred around 170 ms after the stimulus onset. This further corresponds to previous findings that specify the latency for continuous auditory stream processing to be between 120 and 220 ms (Aiken and Picton, 2008; Mesgarani and Chang, 2012; Power et al., 2012; Choi et al., 2014). Considering how these latencies correspond to the latency of N1-P2 auditory evoked potential component, it is possible that, in addition to the activation of attention networks, attended auditory sensory processing largely contributes to successful envelope tracking. This further goes in favor of the hypothesis that sharp auditory events evoking an N1 response, like sentence onsets, drive cortical oscillations to track the continuous speech and thereby facilitate understanding (Doelling et al., 2014).

The EASE from cap-EEG recordings was more closely related to the original attended stream than the EASE from ear-EEG recordings, resulting in lower performance of the cEEGrid. It is unlikely that differences in EEG amplifier technology contributed to this result (De Vos et al., 2014b). Instead, the performance drop could be due to three different factors, which we investigated in this study: the different number of channels, the different sensor locations, and the stronger presence of noise in one of the two systems. These issues will be discussed below.

In a previous study (Mirkovic et al., 2015) we showed that with as few as five EEG sensors, if optimally placed on scalp, decoding accuracy is reliably above chance level. A systematic analysis of single channel contributions to decoding performance revealed that bilateral temporal locations contributed most consistently. Furthermore, several studies revealed that oscillations in the left superior temporal gyrus are coupled to the temporal envelope of attended auditory stream (Kubanek et al., 2013; Vander Ghinst et al., 2016), while functional neuroimaging revealed bilateral anterior superior temporal lobe contributions to sentence comprehension (Humphries et al., 2001). To what extent these processes contribute to adjacent cap-EEG signals is unclear, but the overall pattern of bilateral temporal areas picking-up sentence comprehension related processing seems plausible. Unfortunately, inconspicuous EEG acquisition from multiple sites is not easily possible at the more hairy head locations (Nikulin et al., 2010). However, despite the fact that the spatial coverage of the cEEGrid is not optimal, the captured brain activity is sufficient for successful decoding.

Anticipating that non-optimal channel placement may be more detrimental to decoding performance than the number of channels itself, we used different 16 cap-channel sub-samples to investigate the role of spatial sampling further. With the same number of channels, different layouts resulted in different decoding performance. The best results were achieved when temporal and fronto-central areas were covered, in which case performance did not differ significantly from the high-density configuration. While this analysis also indicated that the cEEGrids were not optimally placed for the task at hand, the ear-EEG signals performed better than cap electrode layouts covering the posterior and/or frontal scalp areas. This indicates that sensor technology differences, such as size and make of electrodes, did not contribute systematically to differences between cap-EEG and ear-EEG. We believe that the spatial location matters for attended speaker decoding more than electrode size or channel number.

We reasoned that one type of artifact in cEEGrid measurements may originate from poor skin-to-sensor adherence in the hair-covered area. Note that, in most individuals, the two top-most cEEGrid channels are located at hairy scalp sites, whereas the other channels are placed at hair-free locations. Despite the tendency to place the cEEGrids identically in each subject, the true positioning and adherence is dependent on the individual differences in skin and hair condition of the participants. For the same reason there is a possibility that the impedance of cEEGrid electrodes located above the ears, where the skin may be obscured by hair and therefore the conductive surface may not be close to the skin, is higher than the impedance on other electrodes. This would generally be reflected in the increase of impedance measured on the respective electrodes compared to other electrodes, and in higher-frequency broadband power, due to noise contributions. However, our analysis did not show any systematic influence of skin-sensor impedance on speech decoding performance, possibly because a high input impedance amplifier was used. Careful skin preparation and appliance of electrolyte gel reduced the electrode impedances well enough.

Due to the cEEGrid placement around the ear, prominent physiological artifacts would be expected to be eye blinks, lateral eye movements and electromyographic activity caused by jaw and head movements. Even though participants were instructed to keep still, there is a chance that these artifacts were more present in ear-EEG signals compared to scalp-EEG signals, and this in turn may have contributed to differences in decoding performance. However, we found no difference in high-frequency power between good and poor performers of the attention task, which renders this interpretation unlikely. Finally, the speech decoding performance was not biased by systematic differences in cEEGrid placement around the ear; individual differences in cEEGrid placement, which could not be avoided due to individual differences in ear size, shape and location, were not related to decoding performance. Hence, the only aspect explaining the lower decoding performance with cEEGrids compared to cap EEG signals appears to be the difference in electrode placement and possibly electrode distance. Aiming for unobtrusive, inconspicuous long-term EEG acquisition (Debener et al., 2015), the cEEGrid is not positioned at optimal scalp sites for attended speaker decoding. Nevertheless, this drawback still allowed above chance-level performance in nearly all participants. Future work aiming at cEEGrid-dedicated signal processing might help to close this gap in performance.

A next generation brain-computer interface designed for the goal of steering hearing devices could benefit from cEEGrid devices, which may be reduced to typical behind-the-ear hearing aid locations in the future. From the user perspective, cEEGrids are more comfortable to wear than EEG caps. Our participants did not report any discomfort when cEEGrids were adequately fitted, which requires keeping a minimum distance between device and the back of the concha. The possibility of long-term measurements in real-life settings (Debener et al., 2015) requires that participants can learn to disregard the presence of EEG technology after a short adaptation period, and the cEEGrids provide this potential. Nevertheless, several obstacles need to be overcome in attended speaker decoding before an integration of state-of-the-art EEG into hearing aid technology could take place. One drawback of current speech decoding is the large amount of data (~30–60 s) needed before a reliable decision can be made. This results in low information transfer rates and a sluggish capturing of attention switches. Faster algorithms based on the state dynamics have recently been developed (Akram et al., 2016) and may alleviate this problem. Finally, progress in decoding-algorithm development appears necessary to facilitate a transition to real-time speech attention decoding.

## Conclusion

In this study we used concurrent ear-EEG and cap-EEG recordings to evaluate the possibility of identifying the attended speaker with flex-printed electrodes placed around the ear (cEEGrid). Our study provides evidence that unobtrusive miniaturized electrodes placed around the ear are sufficient to successfully decode the attended speaker in two-speaker scenarios. The main factor contributing to lower performance of ear-EEG compared to cap-EEG was the spatial location of electrodes placement. More advanced decoding algorithms may allow to combine cognitive state decoding with hearing devices, which would enable these devices to flexibly adjust to a user's listening demands.

## Author contributions

BM, MB, MD, and SD designed the experiment. BM and MB performed data acquisition. BM analyzed the data and wrote the manuscript to which MB, MD, and SD contributed with critical revisions. All authors approved the final version and agree to be accountable for this work.

### Conflict of interest statement

The authors declare that the research was conducted in the absence of any commercial or financial relationships that could be construed as a potential conflict of interest.
